# Comparison of the Effects of Maternal Supportive Care and Acupressure (at BL32 Acupoint) on Labor Length and Infant's Apgar Score

**DOI:** 10.5539/gjhs.v8n3p236

**Published:** 2015-08-18

**Authors:** Marzieh Akbarzadeh, Zahra Masoudi, Najaf Zare, Maryam Kasraeian

**Affiliations:** 1 Maternal-Fetal Medicine Research Center, Department of Midwifery, School of Nursing and Midwifery, Shiraz University of Medical Sciences, Shiraz, Iran; 2 Traditional Medicine and History of Medicine Research Center, Department of Midwifery, School of Nursing and Midwifery, Shiraz University of Medical Sciences, Shiraz, Iran; 3 Department of Biostatistics, School of Medicine, Infertility Research Center, Shiraz University of Medical Sciences, Shiraz, Iran; 4 Maternal-Fetal Medicine Research Center, Department of Obstetrics & Gynecology, Shiraz University of Medical Sciences, Shiraz, Iran

**Keywords:** acupressure, Apgar score, Doula, labor

## Abstract

**Background and Objectives:**

Prolonged labor leads to increase of cesarean deliveries, reduction of fetal heart rate, and maternal as well as infantile complications. Therefore, many women tend to use pharmacological or non-pharmacological methods for reduction of labor length. The present study aimed to compare the effects of maternal supportive care and acupressure (at BL32 acupoint) on labor length and infant's Apgar score.

**Methods:**

In this clinical trial, 150 women with low-risk pregnancy were randomly divided into supportive care, acupressure, and control groups each containing 50 subjects. The data were collected using a questionnaire including demographic and pregnancy characteristics. Then, the data were analyzed using Chi-square test and one-way ANOVA.

**Results:**

The mean length of the first and second stages of labor was respectively 157.0±29.5 and 58.9±25.8 minutes in the supportive care group, 161.7±37.3 and 56.1±31.4 minutes in the acupressure group, ad 281.0±79.8 and 128.4±44.9 minutes in the control group. The difference between the length of labor stages was significant in the three study groups (P<0.001). Moreover, the frequency of Apgar score≥8 in the first and 5^th^ minutes was higher in the supportive care and acupressure groups compared to the control group, and the difference was statistically significant (P<0.001).

**Conclusion:**

Continuous support and acupressure could reduce the length of labor stages and increase the infants’ Apgar scores. Therefore, these methods, as effective non-pharmacological strategies, can be introduced to the medical staff to improve the delivery outcomes.

## 1. Introduction

Delivery pain is an acute pain which rapidly increases and is affected by physiological, psychological, social, cultural, and environmental factors (Leeman et al., 2003). Excessive pain intensifies mother's fear and anxiety during delivery and stimulates sympathetic nervous system. These, in turn, enhance secretion of catecholamines, such as epinephrine and norepinephrine, eventually leading to more pain, prolonged labor stages, and dissatisfaction with the delivery experience (Sercekus & Okumus, 2007). Prolonged labor results in anxiety, fear, and fatigue which play a major role in reduction of mother's self-confidence and self-esteem. Thus, the women experiencing prolonged labor tend to make use of analgesic methods. Prolonged labor also increases the probability of damage, prenatal mortality, utilization of oxytocin, and rate of cesarean and instrumental delivery (Kaptchuk, 2002; May& Elton, 1998; Rabl, Ahner, Bitschnau, Zeisler, & Husslein, 2001). Furthermore, the relationship between chronic stress and delivery outcomes indicates the necessity of interventions for reducing this factor (Fink et al., 2011). Nowadays, various pharmacological and non-pharmacological methods are used for decreasing labor pain. Yet, since pharmacological methods might be accompanied by some complications for both mother and fetus, non-pharmacological ones are more welcomed. Up to now, a large number of non-pharmacological methods have been proposed for reduction of labor pain with acupressure and supportive care being two important ones (Kimber et al., 2008).

Acupressure is a comprehensive treatment method which dates back to 5000 years ago. In this method, similar to acupuncture, specific reflex points on the body are used for treatment. By pressing these points, muscle tension is removed and blood circulation and vital energy are improved (Yang, 2001). Some researchers believe that reduction of pain following stimulation of acupoints is due to the fact that it prevents transfer of pain stimulants and increases the blood endorphin levels (Chung, Hung, Kuo, & Huang, 2003). Park et al. (2003) stated that acupressure increased the intensity of uterine contractions (Park, Cho, Kwon, Ahn, Lim, & Chang, 2003). In the same line, Skilnand et al. (2002) showed that the first stage of labor was shorter among the participants who underwent acupressure (Skilnand, Fossen, & Heiberg, 2002). Overall, various acupoints are employed for induction and control of delivery and BL32 (Ciliao) is one of these points (Cook & Wilcox, 1997).

The other non-pharmacological method used in the current study was supportive care. This method involved continuous presence of doula and provision of psychological support (reassuring, encouraging, and guiding the mother), physical support (palpation, massage, coldness, hotness, hydrotherapy, position change, and movement), informing and guiding the mother, and facilitation of creation of relationship (helping the woman to express her needs) (Simkin & Bolding, 2004). According to most doulas, mothers cannot predict how labor affects them because they do not know about the delivery process and judge themselves negatively (Gilliland, 2011). On the other hand, self-confidence and the ability to adapt with labor are the predictors of labor pain experience. By supportive care, women can successfully cope with labor pain and stress and feel strong and mentally calm (Simkin & Bolding, 2004). In the study by Kennel et al. (1991), doula's continuous support reduced the labor length by 1-2 hours and increased the mother's capability to control laborthereby resulting in a positive delivery experience (Kennel, Klaus, McGrath, Robertson, & Hinkley, 1991).

Considering the effects of prolonged labor on the delivery outcomes, the present study aims to compare the effects of maternal supportive care and acupressure (at BL32 acupoint) on labor length and infant's Apgar score.

## 2. Methods

### 2.1 Study Design

This randomized clinical trial was conducted in the delivery ward of the selected educational center of Shiraz University of Medical Sciences (Shoushtari hospital in Iran) in 2012.

### 2.2 Setting and Sample

Considering d=5, α=0.05, 1-β=0.90, SD=7, and the following formula, a 126-subject sample size was determined for the study (42 in each group). However, due to the possibility of loss, the sample size was increased to 150 subjects (50 in each group):


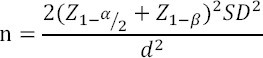


Then, the subjects were selected through simple random sampling and were divided into supportive care, acupressure, and control groups using stratified block randomization. In doing so, a number was randomly selected from the table of random numbers and the researcher moved toward the right or left column or row and wrote the 5 digit numbers down. Since the participants were divided into 3 groups in this study, 3-therapy method was used and classification was performed as follows: A: supportive care group, B: acupressure group, and C: control group. Accordingly, ABC: 1, ACB: 2, BAC: 3, BCA: 4, CAB: 5, and CBA: 6. It should be noted that numbers 0, 7, and 9 were ignored.

### 2.3 Ethical Considerations

Ethical Committee Approval Code in Medical Research,

Shiraz University of Medical Sciences in Iran is CT-P-4985.

### 2.4 Measurements

The inclusion criteria of the study were being primiparous or multiparous, being physically and mentally healthy, having at least diploma, being 18-35 years old, singleton pregnancy, cephalic presentation, gestational age of 37-42 weeks, 4cm dilation, and having at least 2-3 uterine contractions in 10 minutes. After signing written informed consents, the selected women were divided into acupressure, supportive care, and control groups through permuted block randomization. The women with preeclampsia, induced labor, non-cephalic presentation, cephalopelvic disproportion, multiple birth, and those who smoked, suffered from underlying diseases, and were unwilling to take part in the study were excluded from the research.

### 2.5 Procedure

In the supportive care group, the researcher as the doula accompanied the mother since hospitalization up to delivery. Emotional supports during labor included palpation, kindly massaging the mother, and reassuring her. The doula also provided the mother with information about the origin of pain and process of delivery. Besides, physical support included helping the mother to change her position and move during labor.

**Figure 1:**
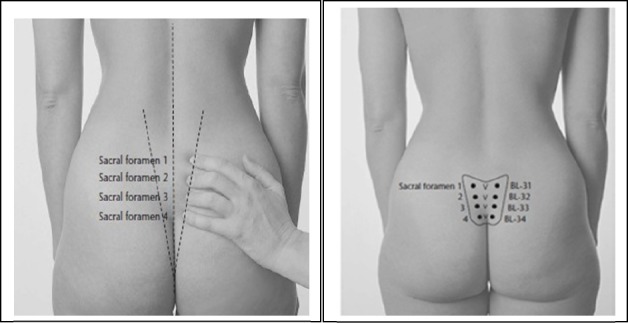
Location of the BL 32 point; Reference: http://acupunctureschoolonline.com/bl-31%E2%80%93bl-34-eight-liao-baliao-acupuncture-points.html

In the acupressure group, in 3-4 and 7-8cm dilation, the participants were located in the appropriate position and BL32 acupoint was pressed. This acupoint is located in the second hole of sacral bone (16). The pressure was continuously and gently applied by both thumbs for 30 minutes (Figure 1). Before sampling, the researcher was trained regarding performance of acupressure by a physical medicine and rehabilitation specialist, so that equal pressure was applied in each performance. After the training, the pressure applied by the right and the left thumb was measured as 1405 and 1277 mmHg, respectively. The pressure was applied by the beginning and stopped at the end of the contractions. Since the interventions were not performed continuously and the researcher took a rest during the contractions, no problems were faced for application of pressure.

The control group only received the hospital's routine care services.

After the interventions, the three study groups were compared regarding the length of the first and second stages of labor as well as the infants’ Apgar scores.

### 2.6 Data Analysis

The data were analyzed using Chi-square test and one-way ANOVA. Post-hoc test was also used to identify the groups with significant differences.

## 3. Results

The results of Chi-square test showed no significant difference among the three groups regarding age distribution and the participants’ mean age (P=0.496). Also, no significant difference was found among the three groups concerning the mothers’ level of education (P=0.584) and occupation (P=0.781). Additionally, the participants’ mean gestational age was 38.9±1.1 weeks and the results of one-way ANOVA indicated no significant difference among the three groups regarding the mean gestational age (P=0.158).

According to the results of one-way ANOVA, the mean length of the first stage of labor was higher in the control group (281.0±79.8) compared to the supportive care (157.0±29.5) and acupressure groups (161.7±37.3). Thus, the control group's mean length of the first stage of labor was 124.0 minutes higher compared to the supportive care group and 119.3 minutes higher compared to the acupressure group, and the difference was statistically significant (P<0.001).

Also, the mean length of the second stage of labor in the control group was 69.5 minutes higher compared to the supportive care group and 72.3 minutes higher compared to the acupressure group, and the difference was statistically significant (P<0.001). According to the results of post-hoc and Least Significant Difference (LSD) tests in the first and second stages of labor, a significant difference was found between the supportive care and the control group as well as between the acupressure and the control group. Overall, the mean length of the first and second stages of labor was lower in the two intervention groups in comparison to the control group. The lowest mean lengths of the first and second stages of labor (157.0±29.5 and 58.9±25.8, respectively) were related to the supportive care group, while the highest mean lengths (281.0±79.8 and 128.4±44.9, respectively) were observed in the control group (Table 1).

**Table 1:** Comparison of the mean duration of the first and second stages of labor in the intervention and control groups

stage of labor	Group	stage of labor	Group (n=50)			P value

			Supportive care	Acupressure	Control	
First stage		Duration (min) M±SD	157.0±29.5	161.7±37.3	281.0±79.8	0.000
		Maximum	210	240	560	
		First stage Minimum	100	60	170	
	CT	Upper limit	165.3	172.3	304.2	
		Lower limit	148.6	151.0	258.8	
		Duration (min) M±SD	58.9±25.8	56.1±31.4	128.4±44.9	
Second stage		Maximum	130	180	200	0.000
		Minimum	20	15	50	
	CT	Upper limit	66.2	65.0	141.1	
		Lower limit	51.5	47.1	115.6	

Significance level: P<0.05. SD: standard deviation.

The results of Chi-square test revealed a significant difference among the three groups regarding the first- and fifth-minute Apgar scores (P<0.001). As Table 2 depicts, the frequency of first-minute Apgar scores<8 in the control group was higher compared to the supportive care and acupressure groups by 46% and 34%, respectively. Also, the frequency of fifth-minute Apgar scores<8 in the control group was higher compared to the supportive care and acupressure groups by 20% and 18%, respectively. The frequency of first-minute Apgar scores≥8 was higher in the acupressure and supportive care groups (86% and 74%) compared to the control group (40%). The frequency of fifth-minute Apgar scores≥8 was also higher in the acupressure and supportive care groups in comparison to the control group (98%, 96%, and 78%, respectively) (Table 2).

**Table 2:** Comparison of the first and fifth minute Apgar scores of the infants in the intervention and control groups

Stage of labor	Group	(n=50)			P value

		Supportive care	Acupressure	Control	
Apgar score after birth	<8	7(14)	13(26)	30(60)	0.000
First minute apgar score	≥8	43(86)	37(74)	20(40)	

Apgar score after birth	<8	1(2)	2(4)	11(22)	0.000
Five minute apgar score	≥8	49(98)	48(96)	39(78)	

Significance level: P<0.05. SD: standard deviation.

## 4. Discussion

The findings of the present study revealed a significant difference between the supportive care as well as the acupressure group and the control group regarding the mean length of the first and second stages of labor (P<0.001). In fact, the mean length of the first and second stages of labor in the control group was respectively 124 and 69.5 minutes higher compared to the supportive care group.

Providing the mother with psychological and emotional support is one of the dimensions of supporting the mother by the doula. In this respect, the findings of the current study were in line with those of the study by Kennel et al. (1991). In that study, women who had experienced delivery acted as doulas and the results indicated a 25% decrease in the labor length in the supported group compared to the control group. A large number of studies have also shown a reduction in the labor length in the supported women compared to those receiving hospital's routine care. This reduction was reported as 2.8 hours in the study by Zhang et al. (1996), 44 minutes in the study by Scott (Scott, Berkowitz, & Klaus, 1999), and 0.58 hours in the study by Hodnett (Hodnett, Gates, Hofmeyr, Sakala, & Weston, 2011). In the study conducted by Campell, the mean length of the first stage of labor was 10.4±4.3 hours in the supported group and 11.7±4.8 hours in the control group. In addition, the mean length of the second stage of labor was 58±51 and 64±57 minutes in the supported and the control group, respectively (Campell, Lake, & Falk, 2006). Similarly, Longer showed that the mean length of labor was 4.56 hours in the supported group and 5.58 hours in the control group (Langer, Campero, Garcia, & Reynoso, 1998). Also, Hofmeyr et al. (1991), Chalmers (1993), Klaus and Kennell (1997), and Pascali-Bonaro (Pascali-Bonaro & Kroeger, 2004) reported that the mean length of labor was lower in the supported group in comparison to the control group. The findings of all the aforementioned studies were in agreement with those of the present study. In all these studies, doulas accompanied the mothers since hospitalization up to the delivery and supported her psychologically. According to these researchers, supporting the mother during delivery can lead to considerable changes in the delivery process, including modification of uterine function, improvement of uterine contractions, creation of effective contractions, and reduction of labor length.

On the contrary to the results of the current study, Bruggemann showed that the mean length of the first stage of labor was 3.4 hours in the supported group and 3.8 hours in the control group (Bruggemann, Parpinelli, Osis, Cecatti, & Neto, 2007). Similarly, Bruggemann and McGrath (McGrath & Kennell, 2008) revealed no significant difference between the intervention (presence of doula) and the control group regarding the mean length of labor. The difference between the results of these studies and the present one might be due to the fact that they were conducted on the individuals from high social levels and the study participants could take their family members to the delivery room either with or without the doula. Therefore, both groups were highly supported and the effect of presence of doula could not be truly investigated.

Overall, presence of doula before the delivery, encouragement, consolation, and palpation of the women, and suggestion of positions which are effective in fetal descent, increase production of oxytocin, enhance women's threshold of pain, and modify delivery pain patterns, thereby decreasing the labor length (Taylor, Klein, Lewis, & Gruenewald, 2000).

According to the western medicine perspective, acupressure can create balance during delivery, reduce delivery pain, and improve the delivery process by increasing the uterine contractions (Beal, 1992). Acupressure is a simple, inexpensive non-pharmacological method for controlling delivery. In general, various acupoints are used for induction and control of delivery (Cook & Wilcox, 1997). In the present study, acupressure was performed at BL32 acupoint and the results revealed a 119.3 minute reduction in the first stage and a 72.3 minute reduction in the second stage compared to the control group, which was statistically significant (P<0.001). In comparison to the supportive care group, the mean length of the first and second stages of labor was higher in the acupressure group; however, the difference was not statistically significant (P>0.005).

Lee et al. compared labor length in acupressure and palpation groups (Lee, Chang, & Kang, 2004). In that study, the active phase of delivery was considered from 3cm dilation to complete dilation. The study results demonstrated that the length of the active phase of delivery was significantly lower in the experimental group compared to the control group, which is consistent with the findings of the current study.

Zeisler stated that acupuncture played a critical role in puberty, acceleration of opening of the cervix, and reduction of labor length. Therefore, due to the positive effects of this method on reduction of labor length, he introduced it as an effective method in controlling labor (Zeisler, Tempfer, Mayerhofer, Barrada, & Husslein, 1998). Reduction of labor length might have resulted from the reduction of pain and its resultant anxiety. In fact, acupressure might lead to release of endogenous opioids and decrease pain and anxiety.

Reduction of delivery pain in the acupressure group can be justified by gate control theory of pain and Melzack's neuromatrix theory. Based on gate control theory of pain, acupressure activates thick nerve fibers and closes the pain gate and in this way, prevents pain transfer. According to this theory, stimulation of skin creates nervous impulses which are transferred to the spinal cord system. These impulses are either inhibited or increased in the spine. The impulses which are transferred by the thick fibers close the pain gate and, consequently, reduce pain. Moreover, stimulation of thick fibers impulses by pressure leads to more closure of the gate (Setax & Pomeranz, 2006).

According to Melzack's theory, pain matrix is composed of three main nervous components, namely sensory way which passes the thalamus and sensory cortex, emotional way which passes the limbic system, and body self-recognition way which includes parietal lobe of cortex. Thus, pain can be sensory, emotional, and cognitive (Chalmers & Wolman, 1993). Hence, in the present study, acupressure affected the sensory way, prevented message transfer to the brain, and reduced the perception of pain.

In contrast, Lawrence mentioned that acupuncture had no effects on reduction of labor length (Lawrence, Lewis, Hofmeyr, Dowswell, & Styles, 2009). The difference between that study and the present one might be due to different definitions of the first stage of labor. In the present study and other similar studies, the first stage of labor began from 3cm dilation to complete dilation. Lawrence, however, considered this stage from the time the number, length, and intensity of contractions were sufficient for opening of the cervix. It is noteworthy that in none of the above-mentioned studies, acupuncture or acupressure increased the first stage of labor. Moreover, not only these non-pharmacological methods did not reduce or inhibit the uterine contractions, but they also sedated the delivery pain and improved the delivery progress.

The findings of the current study showed that the length of the second stage of labor was shorter among the women who received acupressure at BL32 acupoint compared to the control group. In contrast, Lee et al. conducted a clinical trial and indicated no significant difference between the acupressure and the palpation group regarding the mean length of the second stage of labor; i.e., since the complete dilation up to delivery (30.3±22.6 minutes in the acupressure group and 44.8±40.0 minutes in the palpation group). They reported that acupressure was only effective in reduction of the first stage of labor (138.6±62.0 in the acupressure group and 191.2±83.7 in the control group). The difference between Lee's study and the present one might be due to the fact that Lee applied pressure during the contractions. Thus, length of pressure application was different relative to the length of contractions. In the present study, on the other hand, application of pressure was started at the beginning of contractions with similar lengths and intervals for all the participants.

The findings of the current study revealed a significant difference among the three groups regarding the first- and fifth-minute Apgar scores (P<0.001). The frequency of Apgar scores<8 in the first and fifth minutes was higher in the control group compared to the supportive care and acupressure groups. Additionally, the frequency of first- and fifth-minute Apgar scores≥8 was higher in the supportive care and acupressure groups in comparison to the control group.

After 50 years, Apgar scoring system is still the best method for evaluation of newborn infants’ prognosis (Casey, McIntire, & Leveno, 2001). Apgar score has been proved to be the best standard method for evaluation of infants’ health immediately after birth (Jepson, Talashek, & Tichy, 1991). First-minute Apgar score indicated the newborn infants’ need for resuscitation. Besides, fifth-minute Apgar score determines the probability of death or nervous complications more precisely (Roland, 1987). Apgar score, in fact, predicts infants’ chance of survival (Casey et al., 2001). Cunningham et al. demonstrated that prolonged labor was accompanied by Apgar scores<7 due to long labor stages and disruption of delivery phases (Cunningham, Leveno, Bloom, Hauth, Rouse, & Spong, 2010). Furthermore, several researchers have shown that increase in the length of the second stage of labor endangered both maternal and fetal health (Saunders, Paterson, & Wadsworth, 1992) and increased the risk of complications as well as prenatal mortality (Piper, Bolling, & Newton, 1991).

In the study performed by Flan, the mean of first-minute Apgar scores was 8.8 (7-10) in the active group and 7.5 (1-10) in the resting group. Besides, the two groups’ means of fifth-minute Apgar scores were 9.9 (9-10) and 9.4 (7-10), respectively (Flynn & Kelly, 1987). In the same line, Hemminki (Hemminki & Lenck, 1985), Andrews (Andrews & Chrzanowski, 1990), and Ben et al. (2010) revealed that mother's activity during delivery pain reduced the length of labor stages, improved maternal and fetal outcomes, improved infants’ first- and fifth-minute Apgar scores, and reduced the rate of transfer to the neonatal ward. These results were all in agreement with those of the present study. However, Liu (1989) and Stewart (Stewart & Calder, 1984) reported that mother's activity had no effects on improvement of infants’ Apgar scores. The difference between these two studies and the current one might result from the fact that they only investigated physically supporting the mother. In the present study, however, the doula provided the mother with physical support (suggestion of appropriate positions and activities) as well as emotional and mental support which reduced mothers’ anxiety, improved her self-confidence, and decreased labor disorders.

## 5. Conclusion

The findings of the present study showed that supportive care and acupressure reduced the length of labor and increased the infants’ Apgar scores compared to the control group. Therefore, these two non-pharmacological methods which are easy to perform and are not accompanied by any side effects can be employed during labor to achieve better delivery outcomes.
